# Physiochemical basis of human degenerative disease

**DOI:** 10.1515/intox-2015-0003

**Published:** 2015-03

**Authors:** Harold I. Zeliger, Boguslaw Lipinski

**Affiliations:** 1 Zeliger Research and Consulting, Cape Elizabeth, Maine, USA; 2 Harvard Medical School, Joslin Diabetes Center, Boston, Massachusetts, USA

**Keywords:** Alzheimer's disease, cancer, degenerative environmental disease, diabetes, membrane permeability, toxicity mechanism

## Abstract

The onset of human degenerative diseases in humans, including type 2 diabetes, cardiovascular disease, neurological disorders, neurodevelopmental disease and neurodegenerative disease has been shown to be related to exposures to persistent organic pollutants, including polychlorinated biphenyls, chlorinated pesticides, polybrominated diphenyl ethers and others, as well as to polynuclear aromatic hydrocarbons, phthalates, bisphenol-A and other aromatic lipophilic species. The onset of these diseases has also been related to exposures to transition metal ions. A physiochemical mechanism for the onset of degenerative environmental disease dependent upon exposure to a combination of lipophilic aromatic hydrocarbons and transition metal ions is proposed here. The findings reported here also, for the first time, explain why aromatic hydrocarbons exhibit greater toxicity than aliphatic hydrocarbons of equal carbon numbers.

## Introduction

Previously published research has demonstrated the relationship between exposures to exogenous environmental lipophilic chemicals (Ls) and the onset of type 2 diabetes (T2D), cardiovascular disease (CVD), neurodegenerative, neurodevelopmental and adult neurological diseases, cancer and other environmental diseases (Zeliger, 2013, 2013a, 2013b). The onset of these diseases has also been shown to be related to the action of elevated levels of Iron III ions (Fe^3+^) (Lipinski, 2012, 2014; Lipinski & Pretorius, 2012, 2013, 2913a), as well to other transition metal ions (Chen *et al*., 2009; Gauthier *et al*., 2014; Sharma *et al*., 2014). Here we propose a physicochemical mechanism for the onset of environmental degenerative disease that is dependent upon the combined action of aromatic chemical species and transition metal ions (TMs).

We hypothesize that TMs are transported through lipophilic cell membranes via pi-bonding of the TMs to planar aromatic hydrocarbons, thereby raising the levels of the TMs to toxic levels. We further hypothesize that absorption of Ls and TMs need not occur at one time or simultaneously. Rather, the absorptions can occur sequentially over time, with the onset of disease occurring when critical levels of both are reached in the body.

As a consequence of this study, it is explained, for the first time, why aromatic hydrocarbons are more toxic than aliphatic hydrocarbons of equal carbon numbers.

## Methods

The hypotheses presented here are based upon the previous research of the authors, the published literature and an analysis of the bonding in cell membranes and in aromatic hydrocarbon – transition metal ion complexes. The results and discussion sections are combined for clarity.

## Results and discussion

### Cell membrane structure and bonding

Cell membranes are amphiphilic structures composed primarily of a phospholipid bilayer with embedded proteins. The bilayers self assemble to produce hydrophilic heads and hydrophobic (lipophilic) tails which contain embedded proteins. This arrangement allows the membrane to be selectively permeable to ions and other polar molecules, at the head and through ion channels and the passive diffusion of lipophilic species through the tail. The acyl groups in the lipid bilayer vary with cell type and include saturated and unsaturated fatty acid chains with the two layers bonded by covalent disulfide bridges and van der Waals forces (Boggs, 1987). Two types of proteins are embedded in the lipid bilayer. The first, integral membrane proteins, interact primarily in the hydrophobic tails of cell membranes via non-polar bonding. The second, peripheral proteins, interact primarily in the hydrophilic heads of cell membranes, via electrostatic and hydrogen bonds (Lee, 2003; 2004). This variability allows particular cell membranes to selectively permit entry and egress of molecular species essential to the functioning of the these cells. Numerous (in the thousands) of different, unique membrane phospholipid structures have been identified. Each is characterized by the length and degree of saturation of its acyl chain. All vary in membrane thickness, membrane fluidity, curvature and surface charge (Cornell & Separovic, 1983; de Kroon *et al*., 2013; Holthuis & Menon, 2014). The differences in chemical composition and physical structure account for varying permeabilities of membranes, however, as is discussed below, all may be permeated and attacked by lipophilic exogenous chemicals.

### Membrane permeability

Lipophiles are defined as hydrophobic species with octanol:water partition coefficients (Kow) of 2.0 or greater (Zeliger, 2011). Exogenous lipophiles include but are not limited to: aliphatic hydrocarbons, single ring and polynuclear aromatic hydrocarbons (PAHs); phthatlates; bisphenol-A; persistent organic pollutants such as polychlorinated biphenyls (PCBs), organochlorine pesticides (OCs) and polybrominated diphenyl ethers (PBDEs) fire retardants used in clothing; as well as food preservatives and disinfectants such as butylated hydroxytoluene (BHT), butylated hydroxyanisole (BHA) and triclorsan (Zeliger, 2003; 2011).

Exogenous lipophilic chemicals readily penetrate into the hydrophobic tails of phospholipid cell membranes and accumulate there, leading to changes in membrane structure (Endo *et al*., 2011; Librando *et al*., 2003; Campbell *et al*., 2008; McIntosh *et al*., 1980). These changes include: swelling of the membrane lipid bilayer and thereby an increase in membrane surface area; an increase in membrane fluidity; inhibition of primary ion pumps; and an increase in proton and metal ion permeability (Sikkema, 1994; Sikkema *et al*., 1995; Korchowiec *et al*., 2008; Liland *et al*., 2014). These changes bring about inhibition of embedded enzyme activity and the accumulation of exogenous lipophiles in the interior of the affected membranes (Sikkema, 1994; Holthuis & Menon, 2014). Lipophilic chemicals permeate through all cell membrane structures found in the human body, including the blood brain barrier (BBB). These chemicals include PAHs, pesticides, and PCBs as well as low molecular weight hydrocarbons (LMWHCs) (Patel *et al*., 2009; Pardridge, 2012; Qiu *et al*., 2011; Calderon-Garciduenas *et al*., 2008; Gupta, 1999; Seelbach *et al*., 2010).

Lipophiles are used in pharmaceutical formulation to facilitate drug absorption as well as in pesticide formulations to facilitate absorption of hydrophilic neurotoxins through lipophilic membranes (Kitagawa *et al*., 1997; Manganaro, 1997; Patel *et al*., 2009; Pardridge, 2012; Zeliger, 2011). It has been previously reported that lipophiles also facilitate the absorption of organic hydrophilic species that are soluble in the lipophiles, but which hydrophiles alone, are not appreciably absorbed through cell membranes. In such instances, low level toxicity, enhanced toxic effects and attacks on organs that are not attacked by either the lipophilic or the hydrophilic species alone were observed. Those results suggest that mixtures of lipophilic and hydrophilic chemicals have toxicities that are unique compared with the individual species comprising such mixtures (Zeliger 2003; 2011).

### Pathophysiologic role of hydrophilic interactions in biological macromolecules

Spatial structure of major biological macromolecules such as proteins, lipids and nucleic acids is determined by ionic and covalent bonds and by the hydrophobic interactions known to provide the strongest bonds. These type of bonds are the result of inter- and/or intra-molecular interactions between specific chemical groups such as aliphatic and aromatic hydrocarbons. Examples are -CH3 side chains, as in lysine, and aromatic rings, as in tyrosine. Although the true nature of such hydrophobic bonds (HBs) are still not fully understood, they are believed to involve van der Waals forces or similar attractions acting at very close distances (Lodish *et al*., 2007).

In proteins, HBs are responsible for the stabilization of their native conformations by forming intra-molecular bonds within single or multiple polypeptide chains which are buried inside their tridimentional structures. The positions of HBs are determined by the number and locations of disulfides (S–S) which originated from the oxidation of cysteine residues within the polypeptide backbones of the given proteins. Solubilities of plasma proteins are maintained by the presence of hydrophobic groups in the outer shells of protein globules.

Protein denaturation results from a random disulfide exchange reaction leading to the formation of insoluble aggregates (Anfinsen, 1973). A characteristic feature of such aggregates is their insolubility under the physiologic conditions and remarkable resistance to proteolytic degradation, very likely due to their interaction with lipids (Beckerson *et al*., 2015). When one of such aggregates, formed from soluble plasma fibrinogen and termed parafibrin, is generated and circulates, it causes a number of pathologic consequences (Lipinski & Pretorius, 2012). Although hydrophobically bonded protein aggregates cannot be degraded by proteases, their formation can be prevented by a number of mechanisms:

1. Removal of the causative factor(s), such as trivalent iron ion (Fe^3+^) or other transition metal ions (see below), and/or scavenging of hydroxyl radicals formed in the reaction of metal ion with water (Lipinski, 2011).
2. Exposure to amphiphilic substances that displace hydrophobic aggregates from the surfaces of cell membranes (Lipinski, 2014).
3. Maintaining oxidative conditions in the biological milieu by supplying molecular oxygen or other electrophilic species. Such conditions are required to prevent undesirable protein disulfide exchanges that lead to the exposure of intra-molecular hydrophobic epitopes and their subsequent conversion to inter-molecular linkages, with such linkages being responsible for the formation of large insoluble protein aggregates.

One effective electrophilic species is sodium selenite, which is an effective oxidizing agent (Lipinski, 2010). Sodium selenite is particularly effective in the oxidative conversion of protein sulfhydryls into disulfides, as per the following formula:

$$\text{P}-{\left(\text{SH}\right)}_{2}+{\text{SeO}}_{3}^{-2}\to \text{P}-\text{S}-\text{S}-\text{P}+\text{SeO}$$

This formula demonstrates the beneficial health effect of sodium selenite in cancer (Lipinski, 2014), diabetes mellitus (Lipinski & Pretorius, 2012), and in Alzheimer's disease (Lipinski & Pretorius, 2013a). Perhaps the most striking example of the oxidative effects of sodium selenite is its potential inhibition of Ebola and other ennveloped virus infections. The disulfide exchange reaction initiated by viral protein disulfide isomerase (PDI) leads to the formation of hydrophobic spikes in the viral gp120 molecules. These spikes, in turn, penetrate cell membranes in the host cells thus allowing viral entry and multipication (Lipinski, 2014a).

### Iron and other transition metal ion absorption through cell membranes. Metal ion: aromatic interactions

Fe^3+^ is vital for cell homeostasis. As just described, Fe^3+^ can also induce deleterious effects at high concentrations. It is absorbed naturally via the polar heads and ion channels of cell membranes in healthy cells. Other vital transition metal ions are similarly absorbed. There are, however, two other routes by which Fe^3+^ and other transition metal ions may be absorbed. Firstly, cells with badly compromised lipid bilayers may cease to be barriers to hydrophilic species and permit direct passage of metal ions into the interior of cells (Gauthier *et al*., 2014). Secondly, cells that do not have previously damaged lipid bilayers may also be permeated by metal ions via pi-bonding of metals with planar aromatic molecular structures and co-passage of such metal/hydrocarbon complexes through the lipid bilayer. Pi-bonding of metal ions to aromatic ring structures is well established and the passage of metal-PAH complexes through lipophilic cell membranes has been reported in the aquatic environment (Gauthier *et al*., 2014 and the references therein). Support for the hypothesis presented here comes from the known toxic effects of mixtures of metals and aromatic hydrocarbons (Wang *et al*., 2009) as well as from consideration of the absorption of metal ions on planar geometric shapes of PAHs, PCBs, phthalates, bisphenol-A, and single ring aromatics just described (Evans *et al*., 1999; Qu *et al*., 2007; Gauthier *et al*., 2014). Metal ion/arene bonds arise from metal ion/pi-electron complexes which, due to the lipophilicity of the hydrocarbon component of the complex can permeate through the membrane lipid bilayer in a manner analogous to the permeation of lipophile/ soluble hydrophile mixtures, as described above (Zeliger 2003; 2011). Following the penetration of the complex through the lipid bilayer, the complex dissociates, with the metal ions dissolving into the hydrophilic parts of the cell (Gauthier *et al*., 2014). Both absorption routes lead to concentrations of metal ions that are toxic to cells.

### Role of iron and other transition metals in parafibrin formation

**Figure 1:**
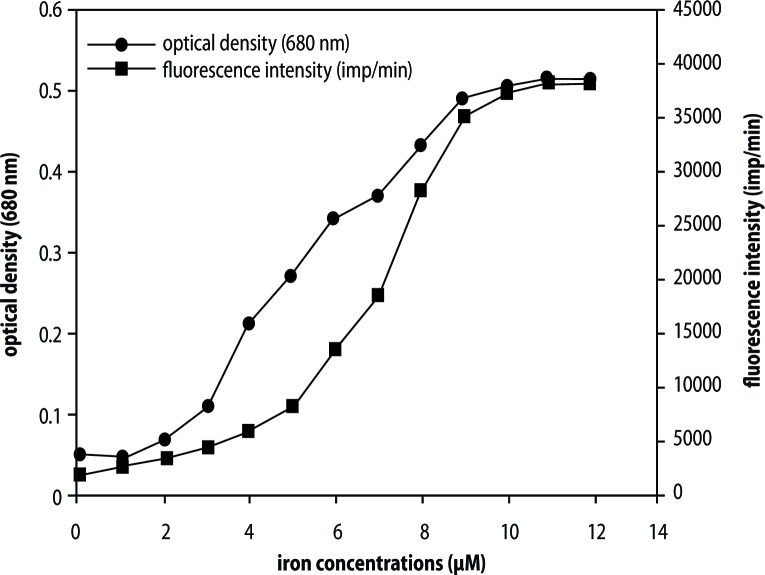
Polymerization of human fibrinogen (optical density), and hydroxyl radical generation (fl uorescence intensity) as a function of iron (ferric chloride) concentration.

It has been previously reported that trivalent iron (Fe^3+^) can activate non-enzymatic blood coagulation resulting in the formation of parafibrin via a hydroxyl radical-catalyzed reaction that generates reactive oxygen species, which, in turn initiates oxidative stress (Lipinski & Pretorius, 2012; Lipinski & Pretorius, 2013; Lipinski & Pretorius, 2013a). As can be seen from Figure 1, ferric ions cause the concomitant generation of hydroxyl radicals (HRs) and polymerization of purified fibrinogen (FBG). (Reprinted from Lipiniski & Pretorius, 2012, with permission).

In addition to iron, other transition metals have also been reported to generate hydroxyl radicals and thereby induce disease in humans. These metals include; silver, copper, vanadium, zinc, cobalt, mercury, lead, cadmium, arsenic, manganese and chromium (Hsu *et al*., 1997; Valko *et al*., 2005; Adlard & Bush, 2006; Park *et al*., 2009; Wang *et al*., 2010; Lipinski & Pretorius, 2013a; Martinez-Finley *et al*., 2013; Xu *et al*., 2014).

### Sequential absorption

Essential metals are continually absorbed and desorbed into and out of healthy cells. Yet, as is the case with iron, excessive concentrations of these can lead to toxic consequences (Pretorius & Lipinski, 2012; Lipinski & Pretorius, 2013; Lipinski & Pretorius, 2013a). Nonessential metals such as cadmium, mercury, lead and chromium are toxic at very low concentration levels and have long half-lives in the human body with that of cadmium being on the order of 20–30 years. These transition metals are not biodegradable and can accumulate in the body with time. (Barbier *et al*., 2005; Tchounwou *et al*., 2012; Rani *et al*., 2014). The association between the sequential exposure to exogenous lipophilic chemicals and the onset of diabetes, cardiovascular disease, neurological disease, cancer and other diseases has been previously reported (Zeliger, 2013; 2013a; 2013b). Exposures to metals (including arsenic, cadmium, mercury lead, nickel, manganese, copper, zinc and chromium) are also known to induce all these diseases via the generation of ROS initiated oxidative stress (Chen *et al*., 2009; Bowman *et al*., 2011; Chen *et al*., 2014, Gauthier *et al*., 2014; Sharma *et al*., 2014). It is also known that metal ions can cross the blood-brain barrier (Bowman *et al*., 2011). It is further hypothesized here that exposures to metals need not occur in one event. Sequential exposures can, over time, generate toxic levels in the body. It is also proposed that toxicity need not be due to a single metal ion species, but that total metal ion load may responsible for toxic effects. This effect is complementary to the sequential absorption of lipophilic species and the reliance on total lipophilic load as indicative of prediction of permeability through lipophilic barriers and the onset of type 2 diabetes, cardiovascular disease, neurological diseases and co-morbidities (Zeliger *et al*., 2012; Zeliger, 2013; 2013a; 2013b; 2014). Also, sequential absorption and accumulation in the body of lipophiles and metal ions means that the body can be exposed to very low levels of toxins and these toxins can accumulate in the body over time until critical levels are reached.

It is also hypothesized that both the lipophiles and the transition metal ions must be present in sufficiently high concentrations to induce disease onset. At times, high concentrations of metal ions alone may present and at times high concentrations of lipophiles alone may present. Since all environmental exposures inevitably include absorption of metals and lipophilic chemicals, it is difficult to ascribe the onset of disease to metal or lipophile alone. Each alone, however, is not believed to induce disease since the body continually metabolizes and/or eliminates both lipophiles and metals. Only when both species are present at critical levels, often involving sequential absorption, is disease triggered. It is also hypothesized that total lipophilic load and total transition metal ion load, irrespective of the chemical nature of the individual species, are critical, since all lipophiles can permeate cell membrane lipophilic bilayers and numerous transition metal ions can pi-bond to a wide spectrum of planar aromatic hydrocarbons. Support for this dual chemical hypothesis comes from a consideration of the published health effects of both lipophilic chemicals and heavy metals. Both species have been shown to cause diabetes, cardiovascular disease, neurological disease (including neurological impairments, neurodevelopmental disorders and neurodegenerative diseases), immunological disease, obesity, cancer and act as endocrine disruptors. It is to be noted that though numerous studies have been published on the effects of a wide spectrum of lipophilic chemicals and heavy metals, no studies have been found that address the effects of mixtures of these species. Yet, it is essentially impossible for an human being living anywhere on the globe today to not be impacted by both lipophiles and transition metals at all times (Bushnik *et al*., 2010; Carvalho *et al*., 2014). Table 2 lists diseases and chemicals causing these as well as representative references of support. Further support for this hypothesis comes from a consideration of the fact that the environmental chemical-induced diseases listed in Table 1 are slow onset diseases, requiring numerous exposures and requiring months to years of exposure prior to their onset.

**Table 1:** Environmental chemical-induced diseases and their causative chemicals.

	CHEMICALS						
DISEASES	POPs	PTL	BPA	PAH	TRM	SRA
T2D	1	1	1	1	1,3–5	1
CVD	6	6	6	6	7	1
NRD	8	8	8	8	7–12	8
IMM	1	6	6	6	7,13	6
CAN	1	14	14	15	7,18	16
OBS	1	1	1	1,17	9	1
END	1	1	1	2,17	2	17

Abbreviations: T2D - type 2 diabetes; CVD - cardiovascular disease; NRD - neurological diseases, including neurological impairments, neurodevelopmental disorders and neurodegenerative diseases; IMM - immunological disease; CAN - cancer; OBS - obesity; END - endocrine disruption; POPs - persistent organic pollutants (including dioxins and furans, PCBs, OCs and PBDEs); PTL - phthalates; BPA - bisphenol A; PAH - polynuclear aromatic hydrocarbons; SRA - single ring aromatic compounds; TRM - transition metals (including silver, copper, vanadium, zinc, cobalt, mercury, lead, cadmium, arsenic, manganese and chromium). References are as follows: 1 - Zeliger, 2013; 2 - Rana, 2014; 3 - Chen *et al.,* 2009; 4 - Kuo, *et al* 2013; 5 - Xu *et al.,* 2013; 6 - Zeliger, 2013a; 7-Jarup 2003; 8 - Zeliger, 2013b; 9 - Rossignol *et al*., 2014; 10 - Kumundi *et al*., 2014; 11 - Fukushima *et al*., 2014; 12 - Lipinski & Pretorius, 2013a; Bai *et al*., 2014; 14 - Sprague *et al*., 2013; 15 - Bostrom *et al*., 2002; 16 - Vizcaya *et al*., 2014; 17 - Scinicariello *et al*., 2014; 18 - Mates *et al*., 2010; 19 - Padilla *et al*., 2010.

### Relative toxicities of aliphatic vs. aromatic hydrocarbons

It is well established that aromatic hydrocarbons are more toxic than aliphatic hydrocarbons of equal carbon number (Perbellini *et al*., 1985; Sato & Nakajima, 1987; Smith *et al*., 2005). This may be seen from a comparison of blood:air partition coefficient (PC) and permissible exposure level (PEL) data. A higher PC indicates a greater propensity to absorb into blood and a lower PEL is indicative of greater toxicity. Table 2 demonstrates this by comparing PC and PEL data for C6–C8 hydrocarbons. As can be seen from the data, aromatic hydrocarbons are more readily absorbed into blood and are more toxic than aliphatic hydrocarbons of equal carbon numbers.

**Table 2:** Blood:air partition coefficient (PC) and permissible exposure level (PEL) data (given in parts per million) for C6-C8 hydrocarbons.

Carbon number	Compound	PC	PEL
6	benzene	7.8	1
	Cyclohexane	1.3	300
7	toluene	15.6	300
	n-heptane	1.9	500
8	p-xylene	28.4	100
	n-octane	3.1	500

The aromatic compound is listed first in each carbon number pair. PEL values are those used by OSHA (OSHA, 2014).

It is hypothesized here that the greater toxicity of the aromatic compounds is due to two factors. Firstly, their higher solubility in blood, from which these compounds can partition into membrane lipid bilayers. Secondly, the transport of transition metal ions dissolved in blood into cell structures via metal:pi-bonding with the planar aromatic species, as described above. Transition metal ions are always present in blood. Iron and some other transition metal ions, are essential and naturally absorbed by the body form healthy foods. Non-essential transition metal ions enter the blood stream via inhalation, ingestion and dermal absorption. Accordingly, when aromatic hydrocarbons are absorbed into the blood, they complex with the transition metals via pi-bonds and the complexed species absorbs through cell membranes, as described above, with resultant higher toxicity than is observed for aliphatic compounds which do not complex with transition metals.

### Parafibrin formation mechanism

The onset of Alzheimer's disease, cancer, type 2 diabetes and cardiovascular diseases, for example have been associated with the excessive Fe^3+^-induced formation of parafibrin (Pretorieus & Lipinski, 2012; Lipinski & Pretorius, 2013; Lipinski & Pretorius, 2013a; Lipinski, 2014). As discussed above, it has been noted that Fe^3+^ activates non-enzymatic blood coagulation via a hydroxyl radical-catalyzed reaction that generates reactive oxygen species, that in turn, initiates oxidative stress, resulting in the formation of parafibrin (Pretorius & Lipinski, 2012; Lipinski & Pretorius, 2013; 2013a). An explanation of why and how this occurs in the presence of excessive iron concentrations has yet to be determined. The literature provides a clue, however. When combined with what is discussed above, a plausible mechanism is suggested. It has been reported that fibrinogen readily absorbs from aqueous media onto microscopic hydrophobic beads dispersed in such media (Retzinger *et al*., 1998). It is suggested here that absorption of exogenous lipophilic species, of the types discussed above, absorb onto fibrinogen. This results in the formation of globules of lipophilic encased fibrinogen which are then acted upon by Fe^3+^ pi-bonded onto the lipophilic molecules to produce parafibrin. Fibrin-like material is known to be present in atherosclerotic plaques (Lipinski & Pretorious, 2013). This demonstrates the propensity of fibrin-like material to bond to lipophilic species and suggests that parafibrin is bonded to lipophilic species that protect the agglomerated mass from enzymatic degradation, as is the case with fibrin itself.

### Disease prevention

When graphed, the release of toxic chemicals to the environment over the past 50 years follows a hyperbolic path. When plotted on the same graph, environmental disease prevalence follows an identical path. There is little doubt that environmental exposures to exogenous toxic chemicals are responsible for the dramatic increase in disease. Genetic and epigenetic factors play major roles in the onset of disease, but these alone cannot account for the dramatic increases in chemically induced environmental diseases that have been observed (Murray *et al*., 2012). The hypotheses presented here are believed to shed new light on the mechanisms underlying the onset of environmental disease. The value of a Mediterranean type diet as a way to prevent environmental disease has been previously reported (Lipinski & Pretorius, 2013a; Schwingshaki & Hoffmann, 2014). In light of what is reported here about the role of transition metals in the induction of disease, the importance of a diet that includes natural chelating agents is underscored. Since environmental exposures cannot always be easily controlled, it is recommended that routine medical examinations include screening blood for total exogenous lipophilic load and for transition metal total load. High levels of either would indicate the risk of disease and the need to take steps to reduce body concentration levels and initiate removal, where possible.

## Conclusions

The following conclusions are drawn:

1. High concentrations of metal ions can enter a cell in two ways; complexed with aromatic molecules or directly if the bilayer is sufficiently damaged.
2. The combination of a lipophilic species and a transition metal ion produce enhanced toxicity to cells when they permeate the lipophilic cell membrane lipophilic barrier together.
3. Aromatic hydrocarbons with planar configurations form pi-bond complexes with transition metal ions that enable the metal ions to permeate into and through membrane lipophilic bilayers.
4. The combination of a lipophilic species and a transition metal ion produce enhanced toxicity to cells when they permeate the lipophilic cell membrane lipophilic barrier either together, or sequentially. The buildup of toxic levels of lipophiles and/or metal ions via sequential absorption strongly suggests that exposures to very low levels of environmental toxicants over time can induce disease and that established permissible exposure levels for toxic chemicals need to be dramatically lowered.
5. Combinations of aliphatic species and metal ions can only act in concert if cell membranes are sufficiently damaged to allow free passage of metal ions. Mixtures of aromatic species and metal ions can be toxic either by co-entry into the cell via pi-bonded complexes, or following separate entry.
6. Aromatic hydrocarbons are more toxic than their aliphatic analogs. This is due to the greater permeability of the aromatic compounds and to their propensity to complex metal ions and transport the ions into cells.
7. Fe^3+^ induced effects that have been reported can be applied to predicting toxic effects of other transition metal ions. It should be noted that there is no mechanism for the elimination of iron from the human body that results in gradual accumulation of this metal with age.
8. The bonding of lipophilic chemicals to fibrinogen potentiates the formation of parafibrin and the prevention of enzymatic breakdown of parafibrin which, in turn leads to disease.
9. The bonding of lipophilic chemicals to parafibrin prevents the enzymatic breakdown of parafibrin.
10. Maintenance of the native structure of proteins is essential for the prevention of degenerative diseases such as cancer, diabetes, cardiovascular, neurological and viral diseases.
11. Lipophilic solvents and/or hydrophobic protein aggregates capable of disturbing the lipid bilayers in the cell membranes can contribute to the progression of degenerative diseases. These pathologic effects can be counterbalanced by reduced exposures to environmental toxicants and by the hydrophilic and oxidative components present in the Mediterranean diet and in certain food supplements.
